# On Tubercles and Phthisis

**Published:** 1844-05

**Authors:** James Black


*On Tubercles and Phthisis.* By JAMES BLACK, M. D.—Considerable difference of opinion still exists among pathologists as to the generation and development of tubercles, as found in the lungs—whether they are the physiological result of a low inflammation, or are a peculiar deposite from the blood, formed by the aggregation of certain morbid or semi-organized molecules, or whether they are an abnormal growth, of the nature of hydatids. Their primary localization is also unsettled. Gerber considers these deposits may arise from any arrestment in the capillaries of the lungs, by which an exudation of the *plasma* or *liquor sanguinis* takes place through the parietes of the vessels into the parenchymal tissue, and so the tubercles are formed—thus making them a mere exudation of albumen, if not of fibrinous globules. Lugol considers their formation to be an *epigenesis*—organized matter capable of nutrition, similar to parasitical growths in other parts, such as in the case of hydatids, intestinal worms, and *pediculi.* All these parasitical complaints are held to be much more frequent in scrofulous habits than in others; and he denies that tubercles begin to soften first in the centre, but equally throughout, being previously quite homogeneous in their texture. As a proof that tubercles are endowed with vascularity, Lugol has met with two instances of hemorrhage in their interior; and, according to his latest opinions, tubercles are no more to be looked upon as devoid of blood-vessels, than the pleura or the white matter of the brain, when congestion or inflammation does not exist; but where vessels are easily detected by the naked eye in all of them, when these phenomena take place. Dr. Kingston also argues for the vascularity of tubercles, and cites, as an analogous proof, the *supposed* non-vascularity of articular cartilage, which he says, has now been proved to be supplied with blood-vessels. In this, he may be said to overlook the ready development of vessels in textures normally corpuscular, or only endowed with cellular permeability. In opposition to these views, many pathologists, especially in this country, consider tubercles to be composed of inorganized matter deposited from the blood on the mucous surface of the air-cells, and, not unfrequently, in the parenchyma of the lungs. It also follows, that as no change can originate in the organized matter itself, so the solidified tubercle can only be affected through the agency of the parts around, or in contact with it. These latter opinions are founded, principally, on the anatomical researches of Dr. Carswell; and Dr. Watson adopts them to their full extent, while he agrees with Lugol, that the formation of tubercles is closely linked with the scrofulous diathesis. While the miliary tubercles are considered by Andral as the pulmonary vesicles rendered hard by chronic *inflammation*, others, following Dr. Carswell, continue to attribute them to a secretion from the blood, not of tubercular matter alone, but also of a portion of deposite. Dr. Watts seems to hold the modified view of tubercles being of an intermediate nature between pus and coagulable lymph, first solidifying, then softening. That tubercles are the pure result of inflammation, is now rarely entertained; but the more modern phraseology is, that they are the result of a perversion of the ordinary process of nutrition; and that the existence of the scrofulous diathesis occasions that to be deposited as inorganizable albumen, which, in a sound state, would have taken on the form of organized fibrine. Dr. Campbell, moreover, in his late *Observations*, infers, with some French physiologists, that the pulmonary capillaries are of smaller dimensions in individuals of strumous habits, than in those of a healthy constitution. This is the predisposition which arrests the imperfectly organized globules in the lungs—a deposition therefore takes place in the pulmonary capillaries, followed in process of time by a reaction, explosive of the disease. M. Louis, in his admirable article on Phthisis, in the *Dictionnaire de Médecine*, lately published, takes a review of all the theories existing on this subject, and considers objectionable that of tubercle being the result of inflammation, or of being nothing but concreted pus, having its seat in the venous capillaries, according to Cruveilhier and Lallemand. He considers the semi-transparent granulation or miliary tubercle as the first degree, or the nucleus which is developed into the crude tubercle; and, on the whole, he seems inclined to attribute some vascular organization to these bodies. The article is a most valuable one, and deserves translation into our language.

Notwithstanding the continued hopeless fatality of tubercu lar phthisis in general, after all the progress that has lately been made into its *genesis* and the intimate analysis of its morbid products, still we may say, we are advancing in the more correct appreciation of those cases, in which a lamentable issue is to take place, through the means of stethoscopic diagnosis. The more perfect knowledge of the predisponent causes and the incipient condition of the disease is, by degrees, leading us to a more scientific application of preventive measures, and, especially, by correcting early aberrations in the nutritive organs. The use also of respirators and of artificial local atmospheres, as recommended by Mr. Jeffreys, if a timely removal to an appropriate climate cannot be accomplished, are also thought more worthy of attention. It would also appear from Liebig's late investigations, that a diminished supply of oxygen from a warm and moist atmosphere, would tend to diminish the amount and severity of pulmonic disease. Mr. Gilbert, in his lately published work, while he considers, like several of his predecessors, the *fons et origo* of the disease to be owing to inorganizable matter being conveyed into the blood from a defective or perverted nutritive process, strongly insists on a judicious regimen and diet in youth, especially where there is a phthisical diathesis in the parents. To detect the earliest invasion of the disease, which is the most difficult point in our stethoscopic diagnosis, he recommends M. Fournet's very discriminative attention to the respiratory murmurs, and especially to those of expiration, which are increased both in duration and intensity, while the inspiratory murmur is increased only in intensity, but diminished in duration.

The occasional discovery of cicatrized cavities in the lungs, such as are related by M. Payan, of Hotel Dieu of Aix, and Drs. Stokes and Graves, has moreover stimulated some to continue the use of empirical remedies, even in the advanced stages. Among these, we have the *proto-iodide of iron* and the *ol. asphalti;* while, in the early periods, we have the pure alkalies highly recommended by Dr. Campbell, and repeated emetics of *ipecacuanha* by Dr. Durant, after the old plan of Dr. Morton. We must not here forget the very elegant arsenical cigarrettoes, invented by M. Trousseau, which in these days of *fumo-mania*, might be easily and effectually adopted, instead of glass inhalers or other stationary apparatus, which are seldom perfectly managed by delicate and nervous patients.

Some of our French brethren are not very sanguine that the light of modern science will add much to the cure of phthisis; and M. Paris, Haute Saone, even says, that since the discovery of auscultation and percussion, the disease is in general more rapidly fatal. He attributes this to an early decided prognosis, whereby the patient is at once put upon a medicated diet, by which the blood becomes sooner impoverished; for he considers the very essence of the disease to be a poverty of nutritious principles in the blood—that its character is chronic, and that its more irregular and rapid progress is owing to the employment of an antiphlogistic treatment, to a dietetic regimen, or to a milk and mucilaginous diet, producing an atony in the digestive organs, and also to the depression of the active moral sentiments. He also cites some cases where a generous diet and dissipation of time, with travelling, produced a cure or lenghtened course of the complaint. I may here remark that some late statistical inquiries have tended to stagger the popular, if not medical opinion, as to the greater prevalence of phthisis in some climates, and among some classes of people, who were thought very obnoxious to the disease. A writer of a very elaborate article in the *London Medical Gazette* has endeavored to show that, from statistical returns, "it is neither inequability of temperature, nor the frequency and magnitude of the variations in temperature to which a country may be subject, that has any effect in the production of phthisis; and Mr. Noble, before the statistical section of the last meeting of the British Association, has deduced some observations, in which some few others concurred, that the deaths from consumption, in the factory district of Manchester, were actually fewer in proportion to the deaths from all causes, than in the agricultural county of Essex, being as four to twenty-one in Essex, and three to nineteen in Manchester; and, finally, he showed that a smaller rate of mortality from this disease, compared with the number of deaths from other causes, existed in the Manchester district, than was to be found in the rural parts of the country.—*Retrospective Address*, in *Trans. of Prov. Med. Association.—Ibid.*

